# Langerhans cell histiocytosis of the suprasellar region: diagnosis based on thyroid cytology

**DOI:** 10.1530/ETJ-24-0011

**Published:** 2024-05-30

**Authors:** Maria Mavromati, Verdiana Caironi, Essia Saiji, Maria-Isabel Vargas, Shahan Momjian, Stephanie Andrade-Lopes, Capucine Gubert, Marco Stefano Demarchi, Ismini Mainta, François R Jornayvaz, Kaveh Samii, Grégoire Stalder, Sophie Leboulleux

**Affiliations:** 1 Department of Endocrinology, Diabetology, Nutrition and Therapeutic Education, Geneva University Hospitals, Rue Gabrielle-Perret-Gentil, Geneva, Switzerland; 2 University of Geneva, Faculty of Medicine, Rue Michel Servet, Geneva, Switzerland; 3 Department of Internal Medicine, Lugano Regional Hospital, Ente Ospedaliero Cantonale, Via Tesserete Lugano, Switzerland; 4 Department of Pathology, Geneva University Hospitals, University of Geneva, Rue Gabrielle-Perret-Gentil, Geneva, Switzerland; 5 Department of Neuroradiology, Geneva University Hospitals, University of Geneva, Rue Gabrielle-Perret-Gentil, Geneva, Switzerland; 6 Department of Neurosurgery, Geneva University Hospitals, University of Geneva, Rue Gabrielle-Perret-Gentil, Geneva, Switzerland; 7 Department of Internal Medicine, Geneva University Hospitals, University of Geneva, Rue Gabrielle-Perret-Gentil, Geneva, Switzerland; 8 Department of Thoracic and Endocrine Surgery, Geneva University Hospitals, Rue Gabrielle-Perret-Gentil, Geneva, Switzerland; 9 Department of Nuclear Medicine, Geneva University Hospitals, Rue Gabrielle-Perret-Gentil, Geneva, Switzerland; 10 Department of Hematology, Geneva University Hospitals, Rue Gabrielle-Perret-Gentil, Geneva, Switzerland; 11 Service and Central Laboratory of Hematology, Lausanne University Hospital, rue du Bugnon Lausanne, Switzerland.; 12 Service of Hematology and Laboratory of Hematology, Institut Central des Hôpitaux, Hôpital du Valais, Av. du Grand-Champsec, Sion, Switzerland

**Keywords:** histiocytosis, thyroid FNA, hypothalamic lesion

## Abstract

Langerhans cell histiocytosis (LCH) may present as unifocal disease of the suprasellar region, with symptoms and signs of hypopituitarism, arginine vasopressin deficiency (AVP-D), and weight gain. Transcranial biopsy is necessary to define diagnosis and guide treatment decisions, but it is associated with significant morbidity. We describe a patient with Hashimoto thyroiditis and a single hypothalamic mass in whom LCH diagnosis was made by thyroid fine-needle aspiration cytology (FNAC) performed despite nonspecific findings in thyroid imaging, on the basis of a slightly elevated [^18^F]-fluorodeoxyglucose (FDG) avidity on PET/CT and volume increase during follow-up.

## Established facts

- Diagnosis of Langerhans cell histiocytosis (LCH) in patients presenting with a single suprasellar lesion is challenging and requires transcranial biopsy, which is associated with increased morbidity.
- Only a few case reports exist on thyroid involvement in LCH, mostly associated with multisystemic disease.

## Novel insights

- The prevalence of thyroid involvement in LCH could be underestimated as clinical and radiological findings are nonspecific.
- Thyroid FNAC is a minimally invasive procedure that can establish LCH diagnosis and provide molecular characteristics to guide treatment. It should be considered as part of the differential diagnosis workup in cases where LCH is suspected.

## Introduction

Pituitary stalk and hypothalamic lesions are rare and may present with rapid weight gain, hypopituitarism, and arginine vasopressin deficiency (AVP-D). Etiology includes inflammatory diseases (such as neurosarcoidosis, histiocytosis, granulomatous, or lymphocytic hypophysitis) and neoplasia (such as craniopharyngiomas, germinomas, or metastasis). Defining the specific cause often requires a transcranial biopsy, which may be associated with considerable morbidity. Diagnostic protocols include screening for extra-cranial involvement (whole-body CT, [^18^F]-fluorodeoxyglucose (FDG)-PET/-CT, bone scintigraphy) and blood and cerebrospinal fluid (CSF) analysis. However, even if the diagnosis is raised after a thorough workup and imaging, histological proof and molecular characteristics of the specific cause are still required to guide treatment (1).

Thyroid involvement in Langerhans cell histiocytosis (LCH) is rare, and co-existence with pituitary disease is only described in case reports (2, 3, 4, 5, 6, 7). Thyroid ultrasound-guided fine needle aspiration cytology (FNAC) can be a noninvasive and useful tool to confirm the diagnosis and prevent unnecessary transcranial biopsy (3, 8). Here we present the case of a young patient with a single hypothalamic mass and no extra-cranial lesions, who was diagnosed with LCH on thyroid cytology despite nonspecific thyroid imaging and the absence of thyroid nodules.

## Case description

A 25-year-old female patient was referred for investigation of a 35 kg weight gain, polyuria, polydipsia, and secondary amenorrhea. She had previously been healthy and was only receiving levothyroxine for Hashimoto thyroiditis. Blood workup confirmed AVP-D (urinary osmolarity: 44 mosm/kg, blood osmolarity: 289 mosm/kg, Na+ 141 mmol/L) and hypopituitarism, with secondary adrenal insufficiency (basal cortisol: 21 nmol/L, normal ACTH), central hypothyroidism (TSH: 0.138 mIU/L, free T4: 9.5 pmol/L), central hypogonadism (estradiol: undetectable, FSH: 1.7 IU/L, LH: 0.2 IU/L), and hyperprolactinemia (102 µg/L). The patient received hydrocortisone and desmopressin followed by an increase in levothyroxine dose, and her general condition improved.

**Figure 1:**
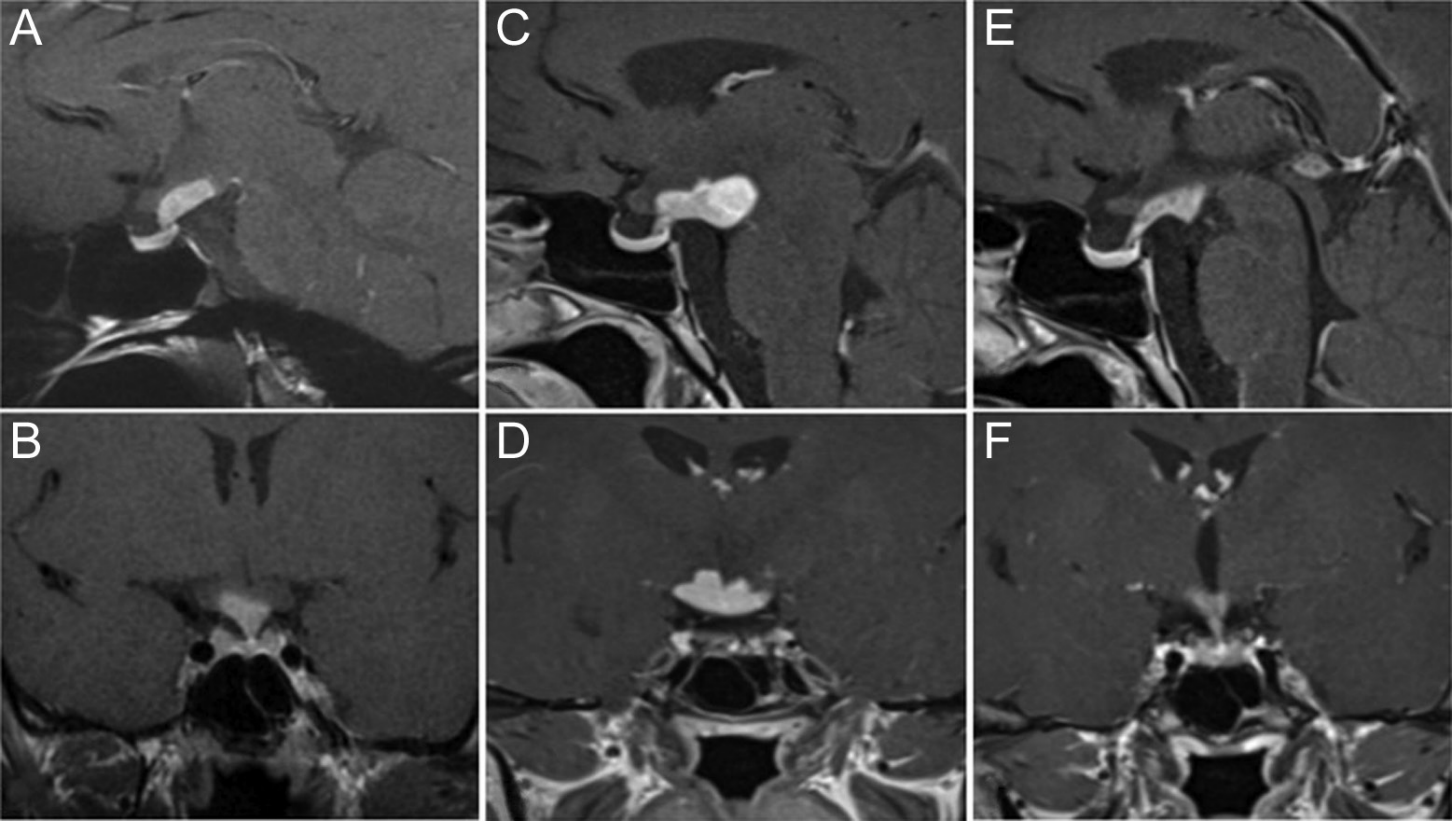
Brain MRI of the suprasellar lesion. Baseline sagittal (A) and coronal (B) enhanced T1 sequences showing a well-delineated hypothalamic-enhanced lesion with recessal involvement, associated with an abnormally thickened pituitary stalk. Follow-up after 12 months of sagittal (C) and coronal (D) enhanced T1 sequences illustrates an increase in the size of the hypothalamic lesion. After six cycles of cladribine treatment, sagittal (E) and coronal (F) enhanced T1 sequences showing a decrease in the size of the lesion.

Brain MRI revealed a 15 mm hypothalamic lesion infiltrating the infundibulum, pituitary stalk thickening, and loss of the posterior pituitary bright spot (Fig. 1A and B). Coronal and sagittal, gadolinium-enhanced, T1-weighted imaging illustrated an intense contrast uptake up to the third ventricle floor and infundibulum. Radiologic features were suggestive of inflammatory disease, although germinoma was not completely excluded at that point.

**Figure 2:**
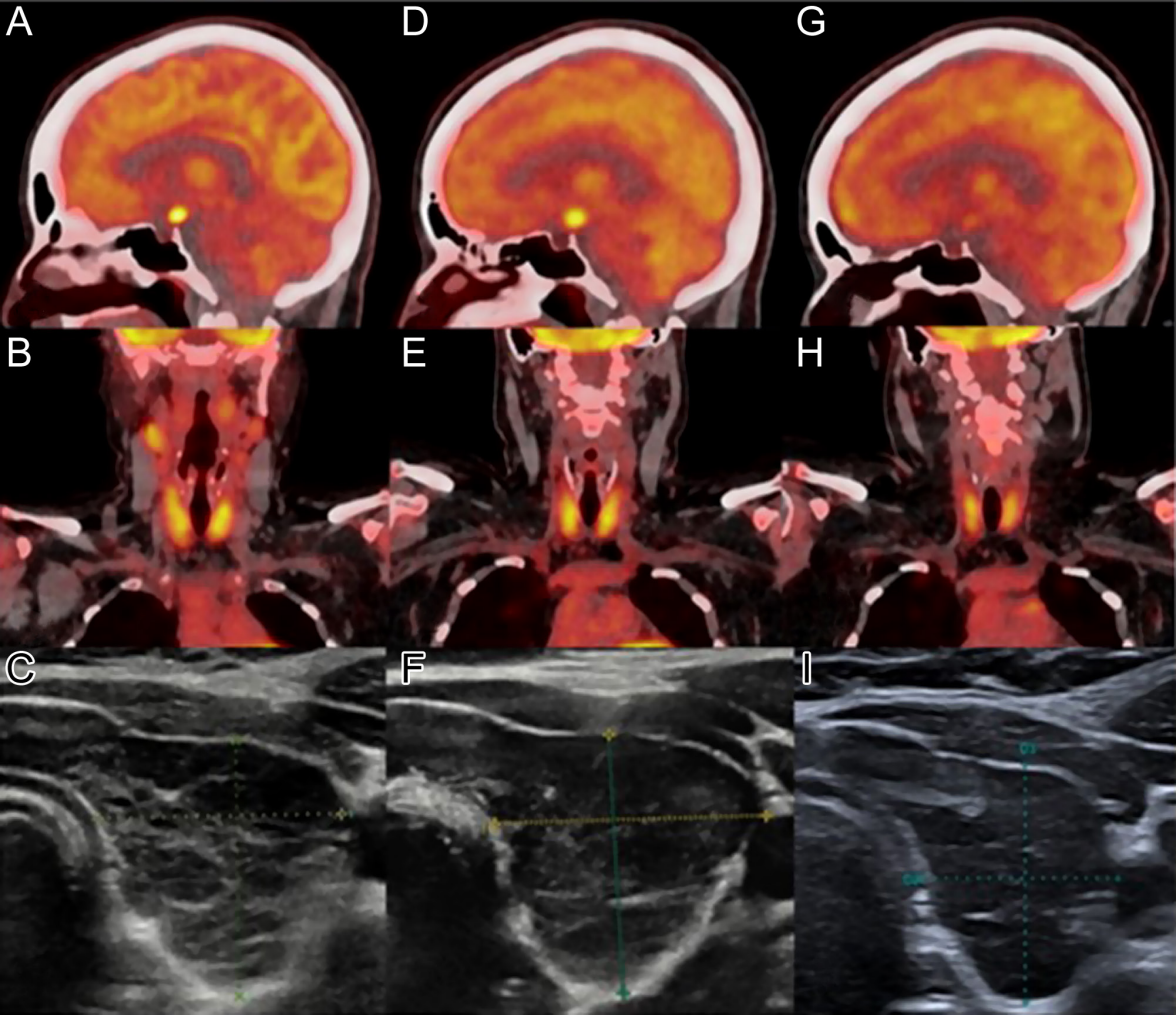
Whole-body FDG-PET/CT and thyroid ultrasound. Sagittal views (A and D) of FDG-PET/CT demonstrating intense FDG uptake of the suprasellar tumor decreasing after cladribine treatment (G). Coronal views (B, E) show moderate, diffuse thyroid hypermetabolism, persisting after treatment (H). Transverse image of the left thyroid lobe (C) upon presentation (thyroid volume 19 mL), on follow-up before specific diagnosis (F) and treatment (thyroid volume 26.7 mL), and after cladribine treatment (I) (thyroid volume 16.2 mL).

Extensive work-up revealed normal β-human chorionic gonadotropin (β-hCG) and alpha-fetoprotein (AFP), as well as normal immunological screening and flow cytometry in the blood and CSF. A potential infectious cause (HIV, hepatitis B and C, Lyme disease, and syphilis) was excluded. Plasma angiotensin-converting enzyme (ACE) and 24-h urine calcium levels were slightly increased (121 IU/L, normal: 20–70 IU/L and 9.6 mmol/24 h, N: 2.5–7.5, respectively). Neurosarcoidosis was considered but finally excluded after CSF analysis (normal white blood cell count, protein levels, and immunoglobulin concentrations). A complete dermatologic examination was unremarkable. Abdominal and chest CT excluded lung, liver, spleen, or gastrointestinal (GI) involvement. FDG-PET/CT (Fig. 2A) showed increased FDG avidity of the suprasellar tumor (SUV 8.7) and slightly increased FDG avidity (SUV 4.1) of the thyroid gland described as related to the known thyroiditis (Fig. 2B) and of inguinal lymph nodes (SUV 3.1). Thyroid ultrasound showed increased thyroid volume (19 mL), with slightly heterogeneous, hypoechoic parenchyma, normal vascularity, and no nodules (Fig. 2C). Anti-thyroperoxidase and anti-thyroglobulin antibodies were increased (276 IU/mL and 64 IU/mL, respectively). The biopsy of inguinal lymph nodes was normal. The patient refused transcranial biopsy for fear of complications. She thus received high-dose steroids (prednisone 1 mg/kg) for 3 months, replaced by methotrexate due to the absence of response.

Consecutive MRIs during a 12-month period showed progression of the lesion expanding to the tuber cinereum and mammillary bodies (Fig. 1C and D). The patient presented new neuropsychiatric symptoms (memory problems, attention deficit, moderate executive dysfunction, depression). As she still refused transcranial biopsy, and histiocytosis was suspected, a new lumbar puncture was performed with a search for mutations in the circulating free DNA (52-gene panel, Ion AmpliSeq Custom Cancer Hotspot Panel IPA). Results showed absence of *BRAF*, *MAP2K1,* or *NRAS* mutation. However, a *RET* exon 11 variant c.1942G>A (p. Val648lle) mutation was found with a variant allele frequency (VAF) of 49%. The same mutation was found in the plasma at a comparable VAF and was thus interpreted as a constitutional variant (polymorphism). Plasma calcitonin levels were normal. PET/CT showed persistence of thyroid and suprasellar FDG avidity (Fig. 2D and E). In the absence of diagnosis and because of an increase in thyroid volume (26.7 mL) without focal lesions, FNAC of both thyroid lobes was performed (Fig. 2F).

**Figure 3:**
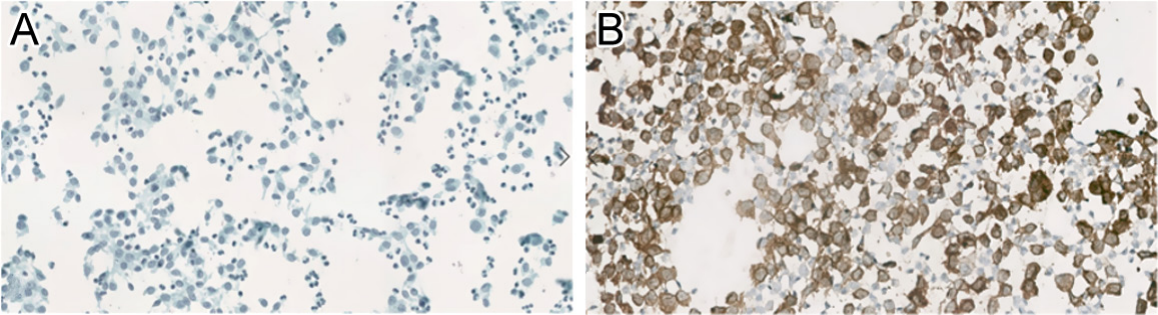
Thyroid FNA cytology. Discohesive epithelioid cells with frequent nuclear grooves and abundant eosinophilic cells in the background (A). Positive immunostaining for CD1a on smear sample (B).

Alcohol-fixed smears with Papanicolaou stain, as routinely performed in our department, showed discohesive and loose clusters of large histiocytes in an inflammatory background rich in eosinophils. The histiocytes had indented and convoluted nuclei with frequent nuclear grooves (Fig. 3A). Some multi-nucleations and rare mitotic figures were seen. No thyroid follicular cells were noted. LCH was confirmed by a diffuse positivity of CD1a (Fig. 3B) and Langerin (CD207), and a weaker expression of S100 protein by immunohistochemistry. Next-generation sequencing (NGS), performed on a cytology sample, revealed the *RET* gene mutation previously described. Thyroid core needle biopsy showed a similar morphologic and immunohistochemical profile.

Given the central nervous system involvement, treatment with subcutaneous cladribine 0.1 mg/kg, 1×/day from day 1 to day 5 for a total of six cycles (28-day cycles) was administered (9). After six cycles, cerebral MRI showed a decrease in the size of the hypothalamic lesion (Fig. 1E and F) with a decrease in FDG uptake on PET/CT (SUVmax 5 versus 10.7), while thyroid uptake was stable (SUVmax 4.9 versus 5.6) (Fig. 2G and H). Thyroid ultrasound showed a decrease in thyroid volume (16.2 versus 26.7 mL) (Fig. 2I). Neuropsychiatric assessment showed a slight improvement in mood and executive functions.

## Discussion

Histiocytosis is rare diseases characterized by hyperactivity or excessive proliferation of histiocytes, a group of cells of the innate immune system with phagocytosis capability (10). More than 100 different subtypes have been described. LCH is so called because the morphology and immunophenotype of pathologic cells resemble Langerhans cells, specialized dendritic cells found in the skin and mucosa. However, LCH is believed to arise from dysregulated myeloid dendritic cell precursors of the bone marrow, and not from Langerhans cells of the skin (11). LCH is characterized by a constant overactivation of the MAPK pathway due to different mutations (e.g. BRAFV600E mutation, MAP2K1). It is currently characterized as an inflammatory neoplastic pathology, with a wide spectrum of clinical manifestations (10).

AVP-D is the most frequent endocrine manifestation associated with LCH and is observed in about 25% of cases upon diagnosis; anterior pituitary dysfunction has been reported in up to 20% of patients, almost always in association with AVP-D (9, 12, 13, 14). Isolated AVP-D can precede LCH diagnosis by several months or years, and radiological findings are often nonspecific (absence of posterior pituitary bright spot, stalk thickening), with only a few patients harboring a suprasellar or hypothalamic mass. Thyroid involvement in LCH is also described as rare and usually related to multisystemic disease (2, 3, 7, 12, 15). In a 2012 review of 65 cases of thyroid LCH, association with a suprasellar or hypothalamic mass was reported only in eight cases, half of which had multisystemic disease where diagnosis is easier (7). Furthermore, in this same review, 24 of 65 cases had isolated thyroid LCH, the majority of which were discovered because of thyroid-related symptoms (such as diffusely enlarged firm goiter), while some cases were incidental FNA diagnoses. Still, we do not know the prevalence of thyroid involvement in patients with confirmed LCH and abnormal but nonspecific thyroid findings since there are no data of routine thyroid cytology in these cases. Clinical features of LCH thyroid involvement may be variable, ranging from diffuse goiter to painless thyroid mass or asymptomatic disease (3, 7, 12). Thyroid function varies from hypothyroidism to euthyroidism and hyperthyroidism (7, 11). Ultrasound findings include a hypoechoic pattern, heterogeneity, and rare calcifications (2, 3, 8, 13, 16). On FDG-PET/CT, LCH thyroid involvement can present as focal or diffuse thyroid hypermetabolism, with a high intensity of FDG uptake in most cases, although moderate metabolism has also been reported (17, 18, 19, 20). However, FDG uptake is not specific, and differential diagnosis from other, more prevalent, thyroid diseases, such as nodules or Hashimoto thyroiditis, based solely on PET/CT results, is difficult (21). Nevertheless, there are no data from routine examination of thyroid functional and radiological status in patients with LCH and no apparent thyroid symptoms, and there is bias in the abovementioned studies, since only cyto-pathologically confirmed thyroid LCH cases were included. No data exist on thyroid imaging changes after LCH treatment neither. In our patient, specific treatment resulted in a decrease of thyroid volume, but FDG uptake remained stable.

In thyroid FNAC, the presence of proliferating Langerhans cells associated with eosinophils is a characteristic cytomorphologic feature of LCH. The expression of specific immunohistochemical markers (CD1a, langerin, and S100 protein) confirms the diagnosis of LCH on cytological samples, sparing a more invasive procedure such as transcranial biopsy. The identification of mutations affecting members of the MAP-kinase pathway in histiocytosis lesions is important both to confirm the diagnosis and to identify possible therapeutic targets (e.g. *BRAF-V600E* mutation, MEK inhibition). Recurrent somatic *BRAFp*.*V600E* mutations are indeed detected in 50–60% of LCH cases and are associated with worse prognoses (22, 23). LCH lacking the *BRAFp.V600E* mutation usually harbor other gene mutations in the MAP kinase signaling pathway.

Transcranial biopsy, whether open or stereotactic, involves some risks. For stereotactic biopsy, the overall morbidity rate was reported to be around 3.5%, but the risk of bleeding, neurological deficit, or death (on average 0.7%) appears to be significantly higher for biopsies in the diencephalic region (24). In our case, thyroid FNA was a safer and more useful alternative, allowing confirmation of diagnosis and offering adequate treatment. In conclusion, thyroid involvement might be more frequent in LCH than believed, as no data exist on real prevalence based on routine thyroid FNA among confirmed LCH cases and abnormal but nonspecific thyroid imaging findings. In the case of suspicion of LCH in a patient with a single intracranial lesion, an extended workup is necessary, especially if a biopsy of the main lesion is not possible due to accessibility difficulties or patient refusal. Whole body FDG-PET/CT, including extremities, is indicated, and a biopsy must be performed in FDG-avid lesions as LCH can affect nearly all organs. A thyroid ultrasound should be performed, especially in the case of increased FDG avidity of the thyroid parenchyma, even in the case of coexisting Hashimoto thyroiditis. As clinical and radiological signs of thyroid involvement in LCH are nonspecific, FNA could be considered as part of the differential diagnosis. Finally, a search for MAPK pathway mutation in peripheral blood and CSF may help non-invasively to orient the diagnosis.

## Declaration of interest

SL is an Editorial Board member of the *European Thyroid Journal*. SL was not involved with the peer-review process of the article on which she is listed as an author. All other authors declare that there is no conflict of interest that could be perceived as prejudicing the impartiality of this case study.

## Funding

This study did not receive any specific grant from any funding agency in the public, commercial, or not-for-profit sector.

## Statement of ethics

Written informed consent was obtained from the patient for publication of her clinical details.

## Author contribution statement

MM: took care of the patient’s follow-up, performed data analysis, drafted and revised the paper. SL: coordinated data analysis and paper conception and revision. VC: took care of the patient and drafted the paper. ES and GS: coordinated diagnosis and molecular data analysis and revised the paper. M-IV and IM: performed and revised imaging and revised the paper. SM, SA-L, CG, MSD, FRJ, and KS: took care of the patient’s follow-up and revised the paper.
